# Fermented foods and preterm birth risk from a prospective large cohort study: the Japan Environment and Children’s study

**DOI:** 10.1186/s12199-019-0782-z

**Published:** 2019-05-01

**Authors:** Mika Ito, Ayako Takamori, Satoshi Yoneda, Arihiro Shiozaki, Akiko Tsuchida, Kenta Matsumura, Kei Hamazaki, Noriko Yoneda, Hideki Origasa, Hidekuni Inadera, Shigeru Saito, Japan Environment and Children’s Study (JECS) Group, Yukihiro Ohya, Reiko Kishi, Nobuo Yaegashi, Koichi Hashimoto, Chisato Mori, Shuichi Ito, Zentaro Yamagata, Hidekuni Inadera, Michihiro Kamijima, Takeo Nakayama, Hiroyasu Iso, Masayuki Shima, Yasuaki Hirooka, Narufumi Suganuma, Koichi Kusuhara, Takahiko Katoh

**Affiliations:** 1 Department of Obstetrics and Gynecology, Faculty of Medicine, University of Toyama, 2630 Sugitani, Toyama City, Toyama 930-0194 Japan; 2 Clinical Research Center, Saga University Hospital, Saga, Japan; 3 Toyama Regional Center for JECS, University of Toyama, Toyama, Japan; 4 Department of Public Health, Faculty of Medicine, University of Toyama, Toyama, Japan; 5 Department of Biostatistics and Clinical Epidemiology, Graduate School of Medicine, University of Toyama, Toyama, Japan

**Keywords:** Preterm delivery, Miso, Yogurt, Cheese, Fermented soybeans, Natto, The Japan environment and Children’s study, JECS

## Abstract

**Background:**

The dietary pattern of pregnant women is known to be associated with preterm birth (PTB). We investigated whether PTB was associated with intake of fermented food by using data from the Japan Environment and Children’s Study.

**Methods:**

From a data set of 103,099 pregnancies, 77,667 cases at low risk for PTB were analyzed. The primary outcome measurements were based on PTB. Fermented food (miso soup, yogurt, cheese, and fermented soybeans) consumption was assessed by using a semi-quantitative food frequency questionnaire.

**Results:**

Intake of miso soup, yogurt, and fermented soybeans before pregnancy significantly reduced the risk of early PTB (< 34 weeks). The adjusted odds ratio (OR) for early PTB in women who had miso soup 1–2 days/week, 3–4 days/week, or ≥ 5 days/week were 0.58, 0.69, and 0.62, respectively, compared with those who had miso soup < 1 day/week (95% confidence interval (CI) 0.40–0.85, 0.49–0.98, and 0.44–0.87). The adjusted OR for early PTB in women who ate yogurt ≥ 3 times/week was 0.62 (95% CI, 0.44–0.87) compared to those who ate yogurt < 1 time/week. The adjusted OR for early PTB in women who ate fermented soybeans ≥ 3 times/week was 0.60 (95% CI, 0.43–0.84) compared to those who ate < 1 time/week. However, the incidence of overall PTB and late PTB (34–36 weeks) was not associated with fermented food intake.

**Conclusion:**

PTB low-risk women with a high consumption of miso soup, yogurt, and fermented soybeans before pregnancy have a reduced risk of early PTB.

**Electronic supplementary material:**

The online version of this article (10.1186/s12199-019-0782-z) contains supplementary material, which is available to authorized users.

## Background

Preterm birth (PTB) is the most significant adverse outcome for maternal and child health in many countries [1–3]. Premature infants are also likely to have long-term impairment and social inequality in their adult life [4] .

There is evidence supporting an association between the dietary pattern of pregnant women and PTB. The Norwegian Mother and Child Cohort Study (*n* = 66,000 women) revealed that a “prudent” dietary pattern, such as higher intake of vegetables, fruits, oils, water, and whole-grain cereals, or a “traditional” dietary pattern, such as potatoes and fish, was associated with a 12% and 9% reduced risk of PTB, respectively [5]. Further, protein-rich and low fat foods were reported to reduce the risk of PTB [6, 7]. In addition to these, fermented foods such as miso soup, yogurt, cheese, and fermented soybeans effect to reduce the risk of PTB, because entero-bacterial flora in PTB cases was different from that in non-PTB cases [8].

Another cohort study in Norway demonstrated an association between high intake of probiotic dairy products and reduced risk of spontaneous PTB [9]. Especially, probiotic intake during the early gestation period was associated with a lower risk of preterm delivery [10]. Kriss et al. reported that the consumption of yogurt by non-obese Mexican women who are classified into low risk of PTB, reduced their risk of PTB [11]. A meta-analysis concluded that probiotic or prebiotic intake during pregnancy did not decrease PTB [12], although the timing and duration of probiotic administration were not consistent across the various studies. A recent study analyzed the difference in outcomes with the timing of probiotic intake and demonstrated that while probiotic intake during early pregnancy reduced PTB, there was no such benefit with intake during late pregnancy PTB [10].

Fermented foods have received attention globally for their role in supporting human health by improving intestinal bacterial flora. Miso is prepared from fermented soybeans. Miso soup, which contains “dashi” stock with miso, is one of the most popular soups in Japan. Fermented soybeans are a traditional Japanese food that have attracted attention for containing protein, isoflavones, and vitamin K. Cheese contains some important nutrients, including calcium and protein, along with vitamin B_12_ and zinc.

In vitro and in vivo studies have suggested that specific fermentation products may actively participate in establishing proper immunological balance [13, 14]. Therefore, we examined whether the intake of fermented foods such as miso soup, yogurt, cheese, and fermented soybeans during early pregnancy period reduces PTB.

## Methods

### JECS population

The current study was based on the Japan Environment and Children’s Study (JECS) data set of 103,099 pregnancies. The JECS is a nationwide, government-funded, multicenter, prospective birth cohort study conducted by the Ministry of the Environment of Japan; details of the study design have been described previously [15, 16], and the characteristics of the project population were reported by Michikawa et al. [16]. The goal of the JECS was to evaluate the effects of different environmental factors on children’s health and development. Written informed consent was obtained from all participating women and their partners.

Pregnant women were recruited from 15 areas in Japan between January 2011 and March 2014. The present study was based on the following datasets: “jecs-ag-20,160,424” (released in June, 2016) and “allbirth_revice001_ver001” (released in October, 2016). All data were obtained from self-reported questionnaires and included information obtained during the first trimester (first questionnaire about pre-pregnancy status) and the second or third trimesters (second questionnaire about prenatal status). Prenatal exposure was assessed by using a semi-quantitative food frequency questionnaire (FFQ) that comprised a list of foods with standard portion (bowl) sizes commonly consumed in Japan [17].

A part of the participants who experienced early PTB did not write in the second questionnaire. We mainly analyzed the first questionnaire data in this study because we want to analyze both early (before 34^+ 0^ weeks) PTB and late (34^+ 0^ to 36^+ 6^ weeks) PTB. The four fermented foods that we analyzed in the first questionnaire correlated with those in second questionnaire (see Additional file 1: Table S1). Significant positive correlations for fermented foods such as miso soup, yogurt, cheese, and fermented soybeans were observed between first questionnaire and second questionnaire.

### Preterm birth (PTB)

The primary outcome, PTB, was defined as birth before gestational week 37^+ 0^. Secondary outcomes were studied by subgrouping PTB as late and early, based on medical (clinical) records.

### Fermented foods (miso soup, fermented soybeans, etc.)

In order to assess the frequency of miso soup intake, the following questions were included in the self-administered questionnaire: “How many times do you have miso soup?” There were six response options: Less than 1 day/month, 1–3 days/month, 1–2 days/week, 3–4 days/week, 5–6 days/week, or every day. In order to assess the frequency of yogurt, cheese, and fermented soybean intake, the following question was included in the self-administered questionnaire: “How many times do you have yogurt (cheese or fermented soybeans)?” There were nine response options: Less than 1 time/month, 1–3 times/month, 1–2 times/week, 3–4 times/week, 5–6 times/week, 1 time/day, 2–3 times/day, 4–6 times/day, or ≥ 7 times/day.

To estimate the risk of PTB based on the intake of each fermented food, we categorized participant responses for consumption of each food type as follows: miso soup, 1–2 days/week, 3–4 days/week, or ≥ 5 days/week and yogurt, < once per week, 1–4 times/week, or ≥ 5 times/week according to previous research [11]. The consumption of cheese and fermented soybeans was categorized as < once/week, 1–2 times/week, or ≥ 3 times/week.

### Statistical methods

Univariate and multivariate logistic analyses were applied to estimate the risk of PTB. We had regarded that two independent PTBs (that is, late PTB and early PTB) existed because they have different risk factors and different profiles. So we did not use multi-nominal logistic regression. We calculated both unadjusted and adjusted odds ratios (OR) with 95% confidence interval (95% CI). Data were expressed as number of PTB cases (2 groups; yes or no) for each category and proportion (%). All statistical analyses were performed by using a statistical software JMP statistical package version 12 and SAS version 9.4 (SAS Institute Inc., Cary, NC). All two-sided *p* values < 0.05 were considered statistically significant.

### Confounding factors for multiple analyses

The confounding factors for multiple logistic models were recognized as follows: age, categorized into 3 groups (≥ 35, 30–34, or < 30); number of previous deliveries (nulliparous: yes/no); pre-pregnancy body mass index (BMI) (kg/m^2^): < 18.5, 18.5–25, or ≥ 25; smoking status (1 = never, 2 = previously smoked but quit prior to current pregnancy, 3 = previously smoked but quit after knowledge of current pregnancy, 4 = currently smoking) for the first trimester (for subsequent trimesters, participants were grouped as smoked or never smoked; educational background (junior high school, high school, technical junior college, technical/vocational college, associate degree, bachelor’s degree, or graduate degree such as Masters/Doctors) was categorized as < 10 years, 10–12 years, 13–15 years, or ≥ 16 years; annual family income (million JPY) was classified as < 2, 2–4, 4–6, 6–8, or ≥ 10; working ≥ 42 h/week calculating from first questionnaire according to previous research [18]: yes/no); and part-timer (yes/no). These confounding factors were reported as risk factors of PTB [2, 19].

The study protocol was reviewed and approved by the Ministry of the Environment’s Institutional Review Board on Epidemiological Studies and by the Ethics Committee of all participating institutions.

## Results

### Participants

**Fig. 1:**
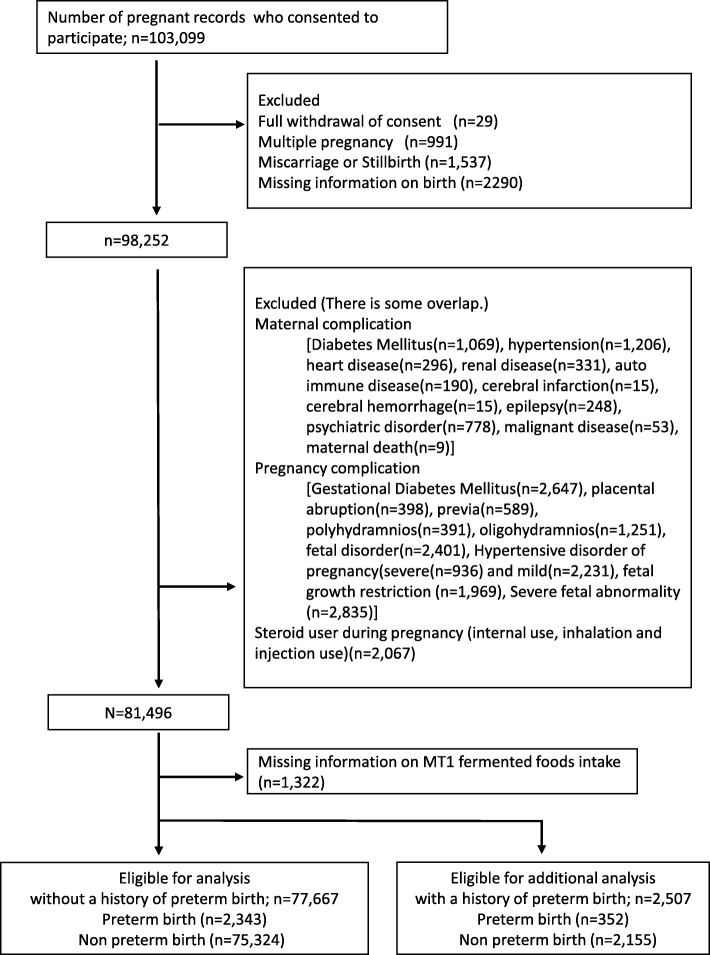
Flow chart showing selection of study subjects

A total of 103,099 pregnancies were analyzed. Initially, the following groups were excluded: participants who withdrew from the study (*n* = 29), multiple pregnancies (*n* = 991), miscarriage or stillbirth (*n* = 1537), inadequate information about birth (*n* = 2290). Further, based on the responses provided in the follow-up questionnaire, the following participants were excluded: known risk factors for PTB and the cause of artificial PTB (hypertension [*n* = 1206], diabetes [*n* = 1069], heart disease [*n* = 296], renal disease [*n* = 331], autoimmune disease [*n* = 190], cerebral infarction [*n* = 15], cerebral hemorrhage [*n* = 15], epilepsy [*n* = 248], psychiatric disorder [*n* = 778], malignant disease [*n* = 53], maternal death [*n* = 9]); steroid use during pregnancy (internal use, inhalation and injection use, *n* = 1516); pregnancy complications (gestational diabetes mellitus [GDM], *n* = 2647); placental abruption (*n* = 398); placenta previa (*n* = 589); polyhydramnios (*n* = 391); oligohydramnios (*n* = 1251); fetal disorder (*n* = 2401); severe (*n* = 936) and mild (*n* = 2231) hypertensive disorders of pregnancy; fetal growth restriction [FGR], *n* = 1969; fetal anomaly (*n* = 2834); and participants with missing information on miso soup, yogurt, cheese or fermented soybean consumption (*n* = 1322). Finally, the remaining 77,667 participants without a history of preterm delivery were included for analysis (Fig. 1). Furthermore, 2507 participants who had a history of preterm delivery that is a great risk for PTB were included for additional analysis.

### Characteristics of the study subjects

The average age at delivery for the 77,667 women in this study was 30.9 ± 5.0 years; mean BMI was 21.1 ± 3.0 kg/m^2^; and mean gestational weeks at delivery was 38.9 ± 1.4 weeks.

The prevalence of PTB in the population was 3.0% (2343/77,667). This prevalence of PTB was lower than in prevalence of PTB in Japan (5.6%), because we have excluded the high risk populations. Older age, low BMI, multiparity, lower education level, and lower income were the significant risk factors for PTB (*p* = < 0.001, < 0.001, 0.022, 0.028, and 0.032, respectively; Table 1). There was not a large correlation coefficient among these foods intake frequencies and between these fermented foods intake and covariates (*r* ≤ 0.32, see Additional file 2: Table S2),

**Table 1:** Characteristics of confounding factors for preterm birth (PTB) cases

	Preterm delivery		Non preterm delivery		*p* value
	*(n = 2343)*		*(n = 75,324)*		
Age, *n* (%)					< 0.001
< 30 years old	867	(37.0)	29,388	(39.0)	
30–34 years old	796	(34.0)	26,841	(35.6)	
≥ 35 years old	680	(29.0)	19,092	(25.4)	
BMI(kg/m2) group, *n* (%)					< 0.001
Leptosome (< 18.5)	488	(20.8)	12,272	(16.3)	
Healthy weight(18.5 to < 25)	1614	(68.9)	56,093	(74.5)	
Obese (≥ 25)	240	(10.3)	6924	(9.2)	
Smoking history, *n* (%)					0.083
Yes	999	(43.1)	30,862	(41.3)	
No	1321	(56.9)	43,931	(58.7)	
Nulliparity, *n* (%)					0.022
Yes	1015	(43.6)	30,927	(41.2)	
No	1314	(56.4)	44,135	(58.8)	
Educational background (years), *n* (%)					0.028
< 10	132	(5.9)	3410	(4.6)	
10–12	748	(33.4)	24,652	(33.2)	
13–15	883	(39.4)	29,886	(40.2)	
≥ 16	476	(21.3)	16,417	(22.1)	
Household income (million Japanese Yen/year), *n* (%)					0.032
< 2	139	(6.6)	3769	(5.4)	
2 to < 4	706	(33.7)	24,147	(34.8)	
4 to < 6	728	(34.7)	22,976	(33.1)	
6 to < 8	303	(14.5)	11,068	(15.9)	
8 to < 10	125	(6.0)	4577	(6.6)	
≥ 10	95	(4.4)	2947	(4.2)	
Working > 42 h/week, *n* (%)					0.691
Yes	481	(20.5)	15,211	(20.2)	
No	1862	(79.5)	60,113	(79.8)	
Part-timer, *n* (%)					0.863
Yes	371	(15.8)	12,027	(16.0)	
No	1972	(84.2)	63,297	(84.0)	

Note: BMI; body mass index before pregnancy

### The difference of fermented food intake state condition by women’s background

Consumption of miso soup was significantly higher in older people, low BMI individuals, non-smokers, multiparous women, and those with high educational background, high household income, not part-timer, and working < 42 h/week (*p* < 0.001, *p* < 0.001, *p* < 0.001, *p* < 0.001, *p* < 0.001, *p* < 0.001, *p* < 0.001 and *p* < 0.001, respectively) (Table 2). Nearly the same results were obtained about consumption of cheese and fermented soybeans. But only yogurt consumption was significantly higher in nulliparous women and working ≥ 42 h/week (*p* < 0.001 and *p* < 0.001, respectively, *not shown in tables*).

**Table 2:** Frequency of miso soup intake and characteristics of confounding factors

	Frequency of intake; miso soup						
	Almost never	1–3 days a month	1–2 days a week	3–4 days a week	5–6 days a week	Every day	*p* value
Age (years), *n* (%)							< .0001
< 30	1522	3604	7592	8279	4829	4393	
	(5.04)	(11.93)	(25.12)	(27.4)	(15.98)	(14.54)	
30–34	1003	2489	6397	7842	5212	4663	
	(3.63)	(9.02)	(23.17)	(28.41)	(18.88)	(16.89)	
≥ 35	671	1631	4217	5523	3837	3872	
	(3.4)	(8.26)	(21.35)	(27.96)	(19.43)	(19.6)	
BMI (kg/m^2^) group, *n* (%)							< 0.001
Leptosome (< 18.5)	492	1237	2927	3492	2430	2180	
	(3.86)	(9.71)	(22.97)	(27.41)	(18.99)	(17.11)	
Healthy weight (18.5 to < 25)	2337	5629	13,526	16,142	10,416	9658	
	(4.05)	(9.76)	(23.46)	(28.00)	(17.98)	(16.75)	
Obese (≥ 25)	366	857	1742	2000	1104	1087	
	(5.11)	(11.98)	(24.34)	(27.95)	(15.43)	(15.19)	
Smoking history, *n* (%)							< 0.001
Yes	1557	3400	7617	8764	5541	4942	
	(4.89)	(10.68)	(23.94)	(27.54)	(17.41)	(15.53)	
No	1602	4259	10,458	12,710	8276	7902	
	(3.54)	(9.42)	(23.13)	(28.12)	(18.31)	(17.48)	
Nulliparity, *n* (%)							< 0.001
No	1595	3707	9974	12,863	8728	8528	
	(3.51)	(8.17)	(21.97)	(28.34)	(19.23)	(18.79)	
Yes	1590	4990	8169	8698	5097	4364	
	(4.98)	(12.5)	(25.6)	(27.26)	(15.97)	(13.68)	
Educational background, years (%)							< 0.001
< 10	239	509	887	887	524	410	
	(6.76)	(14.4)	(25.1)	(27.31)	(14.83)	(11.6)	
10–12	1256	2760	5982	6875	4265	4234	
	(4.95)	(10.88)	(23.58)	(27.1)	(16.81)	(16.69)	
13–15	1123	2883	7075	8727	5675	5256	
	(3.65)	(9.38)	(23.02)	(28.39)	(18.46)	(17.1)	
≥ 16	515	1466	4008	4774	3243	2866	
	(3.05)	(8.69)	(23.76)	(28.3)	(19.22)	(16.99)	
Household income, million Japanese-yen/year (%)							< 0.001
< 2	260	551	997	967	568	561	
	(6.64)	(14.12)	(25.54)	(24.78)	(14.55)	(14.37)	
2 to < 4	1149	2730	6003	6898	4235	3814	
	(4.63)	(11.00)	(24.18)	(27.78)	(17.06)	(15.36)	
4 to < 6	845	2138	5557	6789	4497	3861	
	(3.57)	(9.03)	(22.08)	(28.66)	(18.99)	(16.3)	
6 to < 8	376	1022	2507	3280	2190	1981	
	(3.31)	(9.00)	(22.08)	(28.88)	(19.28)	(17.44)	
8 to < 10	150	403	1145	1313	830	853	
	(3.20)	(8.59)	(24.39)	(27.97)	(17.68)	(18.17)	
≥ 10	84	245	645	819	586	658	
	(2.77)	(8.07)	(21.24)	(26.97)	(19.30)	(21.67)	
Working > 42 h/week (%)							< 0.001
Yes	683	1752	3767	4246	2680	2541	
	(4.36)	(11.18)	(24.04)	(27.10)	(17.10)	(16.22)	
No	2513	5972	14,440	17,399	11,198	10,388	
	(4.06)	(9.65)	(23.32)	(28.10)	(18.09)	(16.78)	
Part-time worker (%)							< 0.001
Yes	506	1389	3017	3437	2103	1931	
	(4.09)	(11.22)	(24.36)	(27.76)	(16.98)	(15.59)	
No	3568	6992	15,509	17,108	10,989	10,542	
	(5.51)	(10.81)	(23.97)	(26.44)	(16.98)	(16.29)	

Note: *BMI* body mass index before pregnancy

### Association between overall PTB and consumption of fermented foods

Table 3 shows the number of cases and the odds ratio (OR) for overall PTB risk as unadjusted and adjusted. Intake of miso soup 1–2 days/week was significantly associated with a lower risk of PTB (unadjusted OR for PTB [95% confidence interval], 0.86 [0.75–0.98]. However, on multivariate analysis, there were no significant associations between risk of PTB and consumption of yogurt, cheese, or fermented soybeans (Table 3).

**Table 3:** Odds ratios (OR) for relationships between preterm birth (PTB) risk and fermented food intake frequency before pregnancy by using logistic regression analysis, Japan Environment and Children’s birth-cohort Study (JECS)

	Cases/all (%)	Unadjusted OR (95% CI)	*p* value	Adjusted OR (95% CI)	*p* value
Miso soup, *n* (%)					
≤ 1 day a week	367/11,008 (3.33)	Reference		Reference	
1–2 days a week	522/18,207 (2.87)	0.86 (0.75–0.98)	0.025	0.87 (0.75–1.00)	0.053
3–4 days a week	643/21,645 (2.97)	0.89 (0.78–1.01)	0.073	0.92 (0.80–1.06)	0.228
≥ 5 days a week	811/26,807 (3.03)	0.91 (0.80–1.03)	0.117	0.93 (0.81–1.06)	0.285
Yogurt, *n* (%)					
< 1 time a week	785/25,354 (3.10)	Reference		Reference	
1–4 times a week	1010/33,129 (3.05)	0.98 (0.90–1.08)	0.742	0.98 (0.89–1.09)	0.735
≥ 5 times a week	548/19,184 (2.86)	0.92 (0.82–1.03)	0.142	0.92 (0.82–1.04)	0.173
Cheese, *n* (%)					
< 1 time a week	1132/37,567 (3.01)	Reference		Reference	
1–2 times a week	737/23,861 (3.09)	1.03 (0.93–1.13)	0.596	1.05 (0.95–1.16)	0.353
≥ 3 times a week	474/16,239 (2.92)	0.97 (0.89–1.08)	0.555	0.98 (0.87–1.10)	0.691
Fermented soybeans, *n* (%)					
< 1time a week	955/30,609 (3.12)	Reference		Reference	
1–2 times a week	813/27,474 (2.96)	0.95 (0.86–1.04)	0.260	0.96 (0.87–1.06)	0.443
≥ 3 times a week	575/19,584 (2.94)	0.94 (0.85–1.04)	0.242	0.96 (0.86–1.08)	0.525

Note: Adjusted OR was estimated by applying multivariable analyses considering the following confounders; mother age, BMI, smoking history, parity, educational background, household income, and working ≥42 h/week. Their categories were shown in method section

### Early PTB (< 34 weeks) or late PTB (34–36 weeks)

Table 4 shows the number of cases and adjusted OR for PTB based on period of gestation. Miso soup intake 1–2 days/week, 3–4 days/week or ≥ 5 days/week significantly reduced the adjusted OR for early PTB (< 34 weeks) (adjusted ORs [95% CI] were 0.58 [0.40–0.85], 0.70 [0.49–0.99], and 0.62 [0.44–0.88], respectively) (Table 4A). The adjusted OR for early PTB (< 34 weeks) in women who had miso soup ≥ 1 day/week was 0.63 compared with those who had miso soup < 1 day/week (95% CI 0.47–0.85; *not shown in tables*). Yogurt intake ≥ 5 times/week and fermented soybean intake ≥ 3 times/week significantly reduced the adjusted OR for early PTB (< 34 weeks) (adjusted OR [95% CI], 0.62 (0.44–0.87) and 0.60 (0.43–0.85), respectively) (Table 4A).

**Table 4:** Odds ratios (ORs) for relationships between PTB risk and fermented food intake frequency before pregnancy by logistic regression analysis, (A)less than 34 weeks, (B) 34 + 0 to 36 + 6 weeks

	(A) Early preterm delivery (< 34 weeks)			(B) Late preterm (34–36 weeks)		
	Cases/All (%)	Adjusted OR (95% CI)	*p* value	Cases/all (%)	Adjusted OR (95% CI)	*p* value
Miso soup, *n* (%)						
< 1 day a week	69/11,008 (0.63)	Reference		298/11,008 (2.71)	Reference	
1–2 days a week	66/18,207 (0.36)	*0.58 (0.40–0.85)*	*0.005*	456/18,207 (2.50)	0.93 (0.79―1.09)	0.354
3–4 days a week	100/21,645 (0.46)	*0.69 (0.49–0.98)*	*0.039*	543/21,645 (2.51)	0.97 (0.83―1.13)	0.660
≥ 5 days a week	102/26,807 (0.38)	*0.62 (0.44–0.87)*	*0.006*	709/26,807 (2.64)	1.00 (0.86―1.16)	0.959
Yogurt, *n* (%)						
< 1 time a week	129/25,354 (0.51)	Reference		656/25,354 (2.59)	Reference	
1–4 times a week	147/33,129 (0.44)	0.83 (0.64–1.09)	0.185	863/33,129 (2.60)	1.01 (0.91―1.13)	0.860
≥ 5 times a week	61/19,184 (0.32)	*0.62 (0.44–0.87)*	*0.006*	487/19,184 (2.54)	0.98 (0.86―1.11)	0.694
Cheese, *n* (%)						
< 1 time a week	176/37,567 (0.47)	Reference		956/37,567 (2.54)	Reference	
1–2 times a week	100/23,861 (0.42)	0.92 (0.70–1.22)	0.570	637 /23,861 (2.67)	1.07 (0.96―1.19)	0.214
≥ 3 times a week	61/16,239 (0.38)	0.71 (0.50–1.00)	0.053	413 /16,239 (2.54)	1.02 (0.90―1.16)	0.738
Fermented soybeans, *n* (%)						
< 1time a week	152/30,609 (0.50)	Reference		803/30,609 (2.62)	Reference	
1–2 times a week	123/27,474 (0.45)	0.89 (0.68–1.16)	0.373	690/27,474 (2.51)	0.97 (0.87―1.09)	0.639
≥ 3 times a week	62/19,584 (0.32)	*0.60 (0.43–0.84)*	*0.003*	513/19,584 (2.62)	1.03 (0.91―1.16)	0.655

Note: Adjusted OR was estimated by applying multivariable analyses considering the following confounders; (A) mother age, BMI, smoking history, parity, previous, educational background, household income, and working ≥ 42 h/week, (B) mother age, BMI, smoking history, parity, previous, educational background, household income, and working ≥ 42 h/week and part-timer. Their categories were shown in the “Methods” section

Cheese intake more than 3 times/week reduced the frequency of early PTB (< 34 weeks), although this was not statistically significant (*p* = 0.053). No association could be identified between the consumption of fermented food and late PTB (Table 4B).

### In women with a history of preterm delivery (additional analysis)

In women with a history of PTB which is known as a major risk factor of PTB, only fermented soybean intake 1–2 times a week significantly reduced the adjusted OR for early PTB which was 0.52 (0.26–0.97) (Table 5). Miso soup and yogurt intake did not reduce the risk of early PTB.

**Table 5:** ORs for relationships between PTB risk and fermented food intake frequency before pregnancy in women who have a history of PTB by logistic regression analysis, (A) less than 34 weeks, (B) 34^+0^ to 36^+6^ weeks (Additional analysis)

	(A) Early preterm delivery (< 34 weeks)			(B) Late preterm (34–36 weeks)		
	Cases/All (%)	Adjusted OR (95% CI)	*p* value	Cases/All (%)	Adjusted OR (95% CI)	*p* value
Miso soup, *n* (%)						
<1 day a week	7/311 (2.25)	Reference		32/311 (10.3)	Reference	
1–2 days a week	14/534 (2.62)	0.99 (0.38–2.62)	0.991	62/534 (11.6)	1.18 (0.74–1.91)	0.481
3–4 days a week	26/725 (3.59)	1.41 (0.59–3.39)	0.441	80/725 (11.0)	1.08 (0.68–1.71)	0.758
≥5 days a week	26/937 (2.77)	1.17 (0.49–2.82)	0.721	105/937 (11.2)	1.03 (0.66–1.61)	0.897
Yogurt, *n* (%)						
<1 time a week	28/844 (3.32)	Reference		87/844 (10.3)	Reference	
1–4 times a week	33/1097 (3.01)	0.89 (0.50–1.57)	0.678	132/1097 (12.0)	1.26 (0.92–1.73)	0.146
≥5 times a week	12/566 (2.12)	0.65 (0.30–1.38)	0.261	60/566 (10.6)	1.08 (0.74–1.57)	0.692
Cheese, *n* (%)						
<1 time a week	33/1161 (2.84)	Reference		122/1161 (10.5)	Reference	
1–2 times a week	26/782 (3.32)	1.40 (0.79–2.49)	0.247	99/782 (12.7)	1.33 (0.98–1.80)	0.065
≥3 times a week	14/564 (2.48)	0.86 (0.42–1.78)	0.686	58/564 (10.3)	0.99 (0.70–1.41)	0.962
Fermented soybeans, *n* (%)						
<1time a week	36/965 (3.73)	Reference		104/965 (10.8)	Reference	
1–2 times a week	21/858 (2.45)	*0.52 (0.28–0.97)*	*0.039*	95/858 (11.1)	1.03 (0.75–1.41)	0.863
≥3 times a week	16/684 (2.34)	0.52 (0.26–1.02)	0.056	80/684 (11.7)	1.03 (0.74–1.44)	0.859

Note: Adjusted OR was estimated by applying multivariable analyses considering the following confounders;(A) mother age, BMI, smoking history, parity, previous, educational background, household income and working ≥42 h/week, (B) mother age, BMI, smoking history, parity, previous, educational background, household income and working ≥42 h/week and part-timer. Their categories were shown in method section

## Discussion

In this study, we examined the relationship between PTB and the consumption frequency of fermented food (miso soup, yogurt, cheese, and fermented soybeans) in Japanese pregnant women determined to be at a low risk for PTB by the JECS. The overall risk of PTB was not reduced in women who consumed fermented foods. However, the risk of early PTB (< 34 weeks) was reduced in women who consumed miso soup at least 1 day/week, yogurt 5 or more times per week, and fermented soybeans 3 or more times per week. Fermented food consumption did not reduce the frequency of late PTB (34–36 weeks) in this study.

In Japan, two birth-cohorts have been conducted before JECS [21, 22], but both studies are locally limited. The study design of JECS is 100,000 participants from 15 regions of Japan reflecting the whole of the Japanese pregnant women more accurately. In these former local studies from Japan, they have assessed the dietary habits of pregnant women, but they did not compare PTB risk with food intake. This study is the first report to show the relationship between PTB risk and food intake frequently from Japan.

Fermented food affects the bacterial flora in the intestines and influences mucosal immunity [14] by increasing secretory type IgA [23] and by inducing anti-inflammatory cells [24] such as regulatory T cells [25]. Yogurt, miso, and fermented soybeans are known to have probiotic effects [26, 27]. Further, the major cause of early PTB (< 34 weeks) is thought to be intrauterine infection caused by ascending vaginal infection. On the other hand, the risk factors for late PTB are mainly environmental and physical factors such as low BMI [28] and part-time work [20], suggesting that the pathogenesis of early and late PTB are different. Therefore, we hypothesized that high consumption of fermented foods may improve immune defenses against infections and reduce the risk of early PTB by improving intestinal bacterial flora.

Several studies have reported on the relationship between PTB and dietary intake. In 2007, the Cochrane Database demonstrated that the use of probiotics effectively reduced bacterial vaginosis during pregnancy [29]. A Mexican group reported that non-overweight women who ate more than 5 cups of yogurt per week had a reduced risk of PTB [11]. In our study, consumption of yogurt > 5 times/week before pregnancy was found to be associated with a reduced risk of early PTB (< 34 weeks) but not overall PTB or late PTB. In contrast, probiotic intake during early pregnancy (not before pregnancy) was associated with a lower risk of PTB, especially late PTB, in a Norwegian cohort [10]. In our study, fermented foods after pregnancy did not reduce late PTB, only cheese intake reduced early PTB risk but miso soup, yogurt, or fermented soybeans did not (Additional file 3: Table S3). These discrepancies may be attributed to differences in the micro-organism content of the fermented food or differences in the bacterial flora or genetic background.

Recent reports suggest that younger Japanese are consuming lesser miso [30]. In JECS study, consumption of miso soup was significantly lower in younger people (Table 2). These changes in eating habits may potentially increase the incidence of PTB. Our result means that health guidance for young women before pregnancy about their eating habits may be effective to prevent PTB.

The beneficial effects of probiotics include symptom alleviation for intestinal diseases (e.g., inflammatory bowel disease [31] and *Clostridium difficile* colitis [32]) and systemic immune diseases (e.g., atopic dermatitis [33], type 2 diabetes [34]), and prophylaxis for acute upper respiratory tract infections [35]. These effects of probiotics on infectious and inflammatory diseases support our hypothesis.

In our study, the distinct effect of fermented food could not be confirmed in women who were at high risk for PTB (Table 5), since the powers of fermented foods are not so effective in high risk women for PTB. The meta-analysis concluded that probiotics during pregnancy have no effect for PTB [12]. All the systematic reviews used in analysis were for PTB high-risk groups—obesity, gestational diabetes, bacterial vaginosis, and so on, and starting period of administration was differrent in each study. Nordquvist et al. reported that probiotics intake from early pregnancy period reduced preterm birth, but probiotics intake from a late pregnancy period did not [10]. The target of this study is PTB low-risk women, and they took probiotics from before pregnancy period. So our study showed that probiotics intake from before the pregnancy period reduced the risk of early PTB (< 34 weeks) in PTB low-risk women, but not reduce the risk for late PTB. Our study showed pre-conceptional fermented food intake might reduce early PTB (< 34 weeks). This is new information. We may recommend taking fermented food before conception.

Our study has a few limitations. First, because the JECS is a cohort study and its data are based on self-administered questionnaires, the current study is subject to possible biases such as withdrawal bias, sampling bias (subjects may or may not have enrolled in the JECS project), selection bias (drop-out or withdrawal from the study), or recall bias (for questionnaires). For example, pregnant women who have high health awareness were expected to participate the JECS study, so it is possible that intake frequency of fermented foods may be higher than the truth by selection bias. Actually, PTB rate in this result, 3.0%, is lower than commonly expected values. In addition, the first questionnaire asked about pre-pregnancy statement, but it was written after pregnancy. So that will not be appropriately reflected in the pre-pregnancy statement by recall bias. Second, the FFQ has not been validated for use with pregnant women. Furthermore, FFQs have several questions and may be a potential source of errors owing to participant assumptions, misunderstanding, or misreading of the questions. Furthermore, as different participants use different sizes of bowls and eat different types of soups, it may have been difficult to calculate and assess intake volumes for completing the FFQs. We therefore analyzed intake frequency, which is least affected by the above-mentioned biases.

## Conclusion

We found an association between the dietary habits of pregnant women and early PTB (< 34 weeks), suggesting that for Japanese women with no risk factors for PTB, high consumption of miso soup, yogurt, and fermented soybeans before pregnancy may decrease their risk of early PTB (< 34 weeks). Further studies are required to clarify lifestyle habits and environment factors that may govern dietary habits.

## Additional files

Additional file 1: **Table S1.** Correlation for fermented foods intake frequency between in first questionnaire and second questionnaire. (DOCX 12 kb)

Additional file 2: **Table S2.** Correlation among fermented foods intake frequency and confounding factors in MT1. (DOCX 16 kb)

Additional file 3: **Table S3.** Odds ratios (ORs) for relationships between preterm birth (PTB) risk and fermented food intake frequency after pregnancy by logistic regression analysis, (A)less than 34 weeks, (B) 34 + 0 to 36 + 6 weeks. (DOCX 17 kb)

## Abbreviations

BMI: Body mass index
CI: Confidence interval
FFQs: Food frequency questionnaires
FGR: Fetal growth restriction
GDM: Gestational diabetes mellitus
JECS: Japan Environment and Children’s Study
OR: Odds ratio
PTB: Preterm birth
