# Supplementary material for: Fermented foods and preterm birth risk from a prospective large cohort study: the Japan Environment and Children’s study
Source: Environ Health Prev Med. 2019 May 1;24:25. doi: 10.1186/s12199-019-0782-z (PMC6492326; doi:10.1186/s12199-019-0782-z)
Supplement: Supplementary file 1 — Table S1. Correlation for fermented foods intake frequency between in first questionnaire and second questionnaire. (DOCX 12 kb) [file 12199_2019_782_MOESM1_ESM.docx]

Supplemental Table 1. Correlation for fermented foods intake frequency between in first questionnaire and second questionnaire

|  | *ρ* | *p-value* |
| --- | --- | --- |
| miso soup | 0.07 | <0.0001 |
| yogurt | 0.58 | <0.0001 |
| cheese | 0.57 | <0.0001 |
| fermented soybeans | 0.67 | <0.0001 |

ρ; Spearman correlation coefficient
