# Supplementary material for: Fermented foods and preterm birth risk from a prospective large cohort study: the Japan Environment and Children’s study
Source: Environ Health Prev Med. 2019 May 1;24:25. doi: 10.1186/s12199-019-0782-z (PMC6492326; doi:10.1186/s12199-019-0782-z)
Supplement: Supplementary file 2 — Table S2. Correlation among fermented foods intake frequency and confounding factors in MT1. (DOCX 16 kb) [file 12199_2019_782_MOESM2_ESM.docx]

Supplemental Table 2. Correlation among fermented foods intake frequency and confounding factors in MT1

Note: ρ; Spearman correlation coefficient, BMI; body mass index before pregnancy
