# Supplementary material for: Fermented foods and preterm birth risk from a prospective large cohort study: the Japan Environment and Children’s study
Source: Environ Health Prev Med. 2019 May 1;24:25. doi: 10.1186/s12199-019-0782-z (PMC6492326; doi:10.1186/s12199-019-0782-z)
Supplement: Supplementary file 3 — Table S3. Odds ratios (ORs) for relationships between preterm birth (PTB) risk and fermented food intake frequency after pregnancy by logistic regression analysis, (A)less than 34 weeks, (B) 34 + 0 to 36 + 6 weeks. (DOCX 17 kb) [file 12199_2019_782_MOESM3_ESM.docx]

| **Supplemental Table 3. Odds ratios (ORs) for relationships between preterm birth (PTB) risk and fermented food intake frequency after pregnancy by logistic regression analysis, (A)less than 34 weeks, (B) 34+0 to 36+6 weeks** | | | | | | | | | | | | | | | | | | | | | | | | | | |
| --- | --- | --- | --- | --- | --- | --- | --- | --- | --- | --- | --- | --- | --- | --- | --- | --- | --- | --- | --- | --- | --- | --- | --- | --- | --- | --- |
|  |  |  |  |  |  |  |  |  |  |  |  |  |  |  |  |  |  |  |  |  |  |  |  |  |  |  |
|  | *(A) Early preterm delivery (< 34 weeks)* | | | | | | | | | | | |  | *(B) Late preterm (34 - 36 weeks)* | | | | | | | | | | | | |
|  | Cases / All ( % ) | | | | Adjusted OR (95% CI) | | | | | |  | *p-value* |  | Cases / All ( % ) | | | | Adjusted OR (95% CI) | | | | | |  |  | *p-value* |
| Miso soup, n (%) |  |  |  |  |  |  |  |  |  |  |  |  |  |  |  |  |  |  |  |  |  |  |  |  |  |  |
| < 1 day a week | 61 | / | 12,931 | (0.47) | reference | | | | | |  |  |  | 334 | / | 12,931 | (2.60) | reference | | | | | |  |  |  |
| 1 - 2 days a week | 64 | / | 18,647 | (0.34) | 0.79 |  | ( | 0.55 | - | 1.15 | ) | 0.216 |  | 452 | / | 18,647 | (2.42) | 0.96 |  | ( | 0.82 | - | 1.11 | ) |  | 0.551 |
| 3 - 4 days a week | 78 | / | 20,471 | (0.38) | 0.83 |  | ( | 0.58 | - | 1.18 | ) | 0.292 |  | 536 | / | 20,471 | (2.60) | 1.04 |  | ( | 0.90 | - | 1.20 | ) |  | 0.627 |
| ≥ 5 days a week | 88 | / | 25,246 | (0.35) | 0.82 |  | ( | 0.58 | - | 1.16 | ) | 0.261 |  | 650 | / | 25,246 | (2.57) | 1.04 |  | ( | 0.91 | - | 1.20 | ) |  | 0.555 |
| Yogurt, n (%) |  |  |  |  |  |  |  |  |  |  |  |  |  |  |  |  |  |  |  |  |  |  |  |  |  |  |
| < 1 time a week | 100 | / | 22,461 | (0.45) | reference | | | | | |  |  |  | 545 | / | 22,461 | (2.42) | reference | | | | | |  |  |  |
| 1 - 4 times a week | 117 | / | 32,310 | (0.36) | 0.85 |  | ( | 0.64 | - | 1.13 | ) | 0.256 |  | 850 | / | 32,310 | (2.60) | 1.11 |  | ( | 0.99 | - | 1.25 | ) |  | 0.067 |
| ≥ 5 times a week | 74 | / | 22,524 | (0.33) | 0.76 |  | ( | 0.55 | - | 1.05 | ) | 0.098 |  | 577 | / | 22,524 | (2.56) | 1.04 |  | ( | 0.91 | - | 1.18 | ) |  | 0.572 |
| Cheese, n (%) |  |  |  |  |  |  |  |  |  |  |  |  |  |  |  |  |  |  |  |  |  |  |  |  |  |  |
| < 1 time a week | 155 | / | 37,484 | (0.41) | reference | | | | | |  |  |  | 956 | / | 37,484 | (2.55) | reference | | | | | |  |  |  |
| 1 - 2 times a week | 92 | / | 23,634 | (0.39) | 0.97 |  | ( | 0.74 | - | 1.27 | ) | 0.799 |  | 590 | / | 23,634 | (2.49) | 1.02 |  | ( | 0.91 | - | 1.13 | ) |  | 0.771 |
| ≥ 3 times a week | 44 | / | 16,177 | (0.27) | **0.67** |  | **(** | **0.47** | **-** | **0.95** | **)** | **0.025** |  | 426 | / | 16,177 | (2.63) | 1.03 |  | ( | 0.91 | - | 1.17 | ) |  | 0.608 |
| Fermented soybeans, n (%) | |  |  |  |  |  |  |  |  |  |  |  |  |  |  |  |  |  |  |  |  |  |  |  |  |  |
| < 1time a week | 130 | / | 31,713 | (0.41) | reference | | | | | |  |  |  | 829 | / | 31,713 | (2.61) | reference | | | | | |  |  |  |
| 1 - 2 times a week | 95 | / | 27,092 | (0.35) | 0.92 |  | ( | 0.70 | - | 1.22 | ) | 1.215 |  | 665 | / | 27,092 | (2.45) | 0.96 |  | ( | 0.86 | - | 1.07 | ) |  | 0.452 |
| ≥ 3 times a week | 66 | / | 18,490 | (0.36) | 0.95 |  | ( | 0.69 | - | 1.29 | ) | 1.288 |  | 478 | / | 18,490 | (2.58) | 1.03 |  | ( | 0.91 | - | 1.16 | ) |  | 0.621 |
| Note: Adjusted OR was estimated by applying multivariable analyses considering the following confounders; (A) mother age, BMI, smoking history, parity, previous, educational background, household income and working ≥ 42hr/week, (B)mother age, BMI, smoking history, parity, previous, educational background, household income and working ≥ 42hr/week and part-timer. Their categories were shown in method section. | | | | | | | | | | | | | | | | | | | | | | | | | | |
|  |  |  |  |  |  |  |  |  |  |  |  |  |  |  |  |  |  |  |  |  |  |  |  |  |  |  |
|  |  |  |  |  |  |  |  |  |  |  |  |  |  |  |  |  |  |  |  |  |  |  |  |  |  |  |
